# Geriatric nutritional risk index as a prognostic factor in patients with hepatocellular carcinoma following transarterial chemoembolization: A retrospective study

**DOI:** 10.1097/MD.0000000000032322

**Published:** 2022-12-23

**Authors:** Youjiao Si, Peng Xu, Aihua Xu, Peiyuan Wang, Kaikai Zhao

**Affiliations:** a Department of Radiology, Shandong Cancer Hospital and Institute, Shandong First Medical University and Shandong Academy of Medical Sciences, Jinan, China; b Department of Radiology, Yantai Affiliated Hospital of Binzhou Medical University, Yantai, Shandong, China; c Department of Radiation Oncology, Shandong Cancer Hospital and Institute, Shandong First Medical University and Shandong Academy of Medical Sciences, Jinan, China.

**Keywords:** chemoembolization, geriatric nutritional risk index, hepatocellular carcinoma, prognosis, therapeutic

## Abstract

The geriatric nutritional risk index (GNRI) has been shown to be associated with the prognosis of cancer patients except for hepatocellular carcinoma (HCC) patients after transarterial chemoembolization (TACE). Our aim is to examine the association between the GNRI and long-term prognosis in patients with HCC who underwent TACE. Patients with HCC who underwent TACE were enrolled. The relationship between the patient characteristics and GNRI were compared, and the independent prognostic factors were investigated. Nomogram performance was assessed via the concordance index (C-index) and calibration plots. Decision curve analysis (DCA) was performed to evaluate the net benefit of the nomogram. A total of 235 patients met the inclusion criteria. Compared with the parameters of the high GNRI group, low GNRI was significantly associated with hypertension, ascites, body mass index, tumor size, anemia, Child-Turcotte-Pugh class. The univariate analysis demonstrated that overall survival (OS) was inferior when GNRI < 98, tumor size ≥ 5cm, vascular invasion, alpha-fetoprotein level ≥ 400, Barcelona clinical liver cancer stage B to C and TACE times < 3. The multivariate analysis revealed that GNRI < 98, tumor size ≥ 5cm, tumor number ≥ 2, alpha-fetoprotein level ≥ 400 and TACE times < 3 were independent predictors of a poor OS. In the validation step, OS was shown to be well calibrated (C-index = 0.724), and a satisfactory clinical utility was proven by DCA. Low GNRI score was associated with a shorter OS in patients undergoing TACE.

## 1. Introduction

Hepatocellular carcinoma (HCC) is the second cause of cancer-related deaths globally and has an incidence of approximately 8,50,000 new patients every year,^[1]^ with over 50% of the new patients occur in China.^[2]^ Risk factors for developing HCC are well known, including hepatitis B and C virus infection, alcohol intake and ingestion of the fungal metabolite aflatoxin B1,^[1]^ often developed either from intrahepatic metastasis or multicentric occurrence, which is a distinct feature of HCC.^[3]^ Hence, most patients lose the opportunity to receive liver transplantation, surgical treatment, or radiofrequency ablation. Chemoembolization has survival benefit in asymptomatic patients with multifocal disease without vascular invasion or extrahepatic spread,^[4]^ which is an efficacy and safety treatment means.

The majority of HCC patients are not only affected by the disease itself but do also suffer from chronic liver disease.^[5]^ Several factors impact on the prognosis of patients with HCC, including tumor itself, liver function, performance status and other comorbidities.^[5]^ The nutritional status is of high significance for the patients’ performance status, the tolerance of tumor-targeting therapy and the prognosis of cancer of any type and is specially referenced in HCC. Geriatric nutritional risk index (GNRI), a new prognostic nutritional index, has been proposed for evaluation of at risk, in-hospital, elderly patients with malignant tumors, especially in tumors of the digestive system, such as head and neck cancer,^[6]^ esophageal squamous cell carcinoma,^[7]^ gastric cancer^[8]^ and colorectal cancer.^[9]^ Li et al found that preoperative lower GNRI value was associated with worse overall survival after hepatectomy in elderly HCC patients,^[10]^ but the relationship between GNRI and prognosis in patients with HCC after transarterial chemoembolization (TACE) has not yet been reported. In the present study, the relationship between GNRI and long-term prognosis for patients with HCC underwent chemoembolization was investigated and a survival prediction nomogram was formulated, and its performance was evaluated.

## 2. Methods

### 2.1. Patients

From January 2014 and December 2018, 235 patients with HCC who underwent TACE were retrospectively enrolled and evaluated from a single-center. The inclusion criteria were as follows: patients were diagnosed with HCC based on the criteria of the American Association for the Study of Liver Diseases; no main portal vein trunk involvement or extrahepatic metastasis; Child-Turcotte-Pugh (CTP) functional class A or B. The exclusion criteria were as follows: large volume of ascites (grade 2 or 3^[11]^); Barcelona clinical liver cancer (BCLC) stage D; received other treatment before or after TACE, such as radiofrequency ablation or radiotherapy; combined with other tumors. In addition, patients with minimal ascites that could only be detected by sonography or computed tomography scans were included in this study, which was deteced in the pelvis and/or hepatorenal angle only.^[12]^ This study was approved by the ethics committee of our institution. Since this was a retrospective study, the requirement to obtain informed consent was waived.

### 2.2. Clinical definitions

The GNRI formula used was as follows: GNRI = 1.487 × serum albumin (g/L) + 41.7 × present/ideal body weight (kg).^[13]^ Blood samples were collected before TACE within 1 week. The ideal weight was calculated from the Lorenz equation, as follows: For males: Height-100- [(Height-150)/ 4]. For females: Height- 100- [(Height-150)/ 2.5]. The patients were classified as having risk (GNRI < 98) and normal level (GNRI ≥ 98) according to previous reports.^[10]^ Anemia defined as a hemoglobin concentration < 120 g/L for males and < 110 g/L for females, in accordance with the Chinese criteria.^[14]^ The BCLC staging system was used as HCC staging criteria.^[15]^ Body mass index (BMI) was calculated as body weight (kg) divided by height^2^ (m^2^), and patients were classified as obese (BMI > 24) and non-obese (BMI ≤ 24) according to Chinese guidelines.^[16]^

### 2.3. TACE treatment

The equipment for hepatic artery angiography was an Artis Zee Floor (Siemens, Germany). At least 2 doctors with more than 5 years of experience performed the operation. Briefly, TACE was performed through a right common femoral arterial approach. The blood supply of the tumor was assessed by using a nonionic contrast medium (Omnipaque, 15 g/50 mL, GE). For chemoembolization, oil emulsions were prepared by mixing 10 to 20 mg doxorubicin hydrochloride (Lunan Pharmaceutical Co., Ltd.) and 10 to 20 mL iodized oil (Yantai Luyin Pharmaceutical Co., Ltd.), according to the tumor volume. The feeding arteries were initially embolized by injecting the prepared oil emulsion and then completely embolized with absorbable gelatin sponge pledgets. Finally, hepatic arteriography was performed to evaluate tumor devascularization. TACE was repeatedly performed if residual viable tumor was confirmed. Patients received repeated TACE when they were assessed failed of therapy according to JSH criteria.^[17]^

### 2.4. Follow-up

Computed tomography scans or magnetic resonance imaging was performed after TACE every 2 to 3 months, and TACE was performed again if tumors with incomplete embolization. Overall survival (OS) was described as the interval between the day of diagnosis and death or the last follow up. Patients were considered censored if without end events at the end of the study. Follow-up was conducted until October 2020.

### 2.5. Statistical analysis

The *χ*^2^ test or Fisher’s exact test was used for categorical data. Survival was estimated using the Kaplan-Meier method and log-rank test. Univariate and multivariate Cox regression hazards models were performed in order to evaluate the prognosis factors on OS. A nomogram was formulated based on the results of multivariate analysis, variables with *P* < .2 in the multivariate analysis were included in the nomogram model. Afterward, the validation was conducted by using concordance index (C-index), calibration curve (1000 bootstrap resamples), and decision curve analysis (DCA). *P* < .05 was considered statistically significant. SPSS 22.0 (IBM Corporation) and R software (version 3.5.3) were used for statistical analysis.

## 3. Results

### 3.1. Patient characteristics

**Figure 1.**
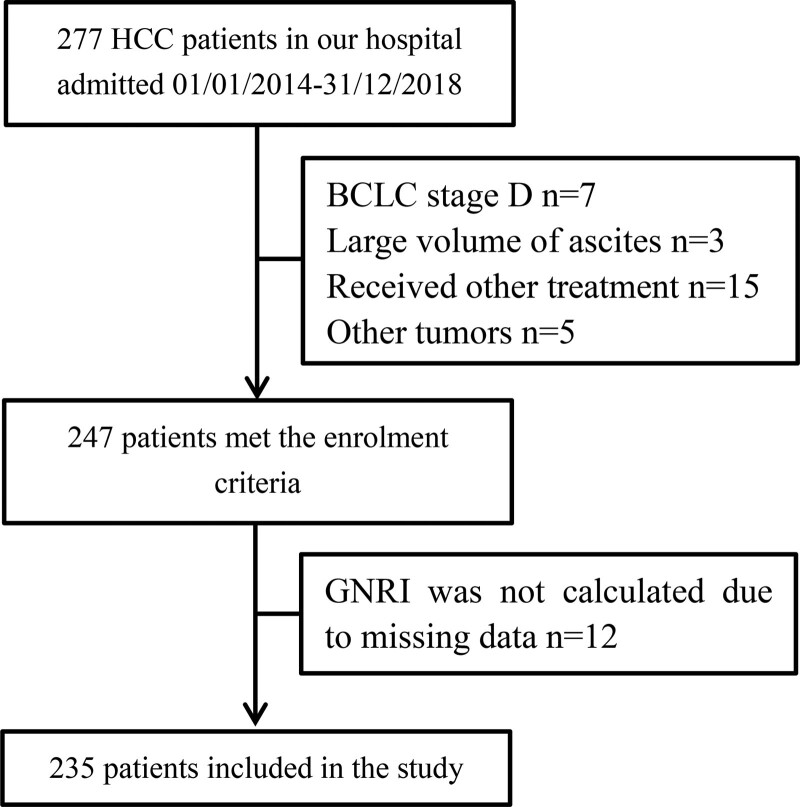
Flowchart for the study.

A total number of 235 patients with HCC undergoing TACE were assessed. The flow chart diagram is presented in Fig. 1. The baseline characteristics of the patients are listed in Table 1. There are 190 (80.9%) males and 45 (19.1%) females. The age of the patients was 59.1 ± 10.4 years for males and 61.2 ± 11.1 years for females. The average total tumor size was 6.4 ± 4.2 centimeters. In total, 57 (24.3%) patients were BCLC A and 178 (75.7%) patients were BCLC B or C. 68 (28.9%) patients had multiple tumors. 11 (4.7%) patients had vascular invasion and 66 (28.1%) patients had anemia. 156 (66.4%) patients had CTP class A and 79 patients had CTP class B, respectively.

**Table 1:** The relationship between the GNRI and the clinicopathological characteristics.

Variables	GNRI < 98	GNRI ≥ 98	*P* value
	n = 111,47.2%	n = 124,52.8%	
Age (yrs)			.152
<65	69	88	
≥65	42	36	
Sex			**.038**
Male	96	94	
Female	15	30	
Hypertension			**.049**
Yes	12	25	
No	99	99	
Diabetes mellitus			.183
Yes	16	11	
No	95	113	
Ascites			**.048**
Yes	9	3	
No	102	121	
BMI			
<24	80	36	**<.001**
≥24	21	88	
Tumor size (cm)			**.016**
<5	40	64	
≥5	71	60	
Tumor number			.541
<2	81	86	
≥2	30	38	
Vascular invasion			.264
Yes	7	4	
No	104	120	
AFP level(ng/mL)			.252
<400	71	88	
≥400	40	36	
Anemia			**<.001**
Yes	49	17	
No	62	107	
CTP class			**<.001**
A	51	105	
B	60	19	
BCLC stage			.232
A	23	34	
B-C	88	90	
TACE times			.065
<3	91	89	
≥3	20	35	

AFP = alpha-fetoprotein, BCLC = barcelona clinical liver cancer, BMI = body mass index, CTP = child-turcotte-pugh, GNRI = geriatric nutritional risk index, TACE = transarterial chemoembolization.

### 3.2. Relationship between the Patient Characteristics and GNRI

**Figure 2.**
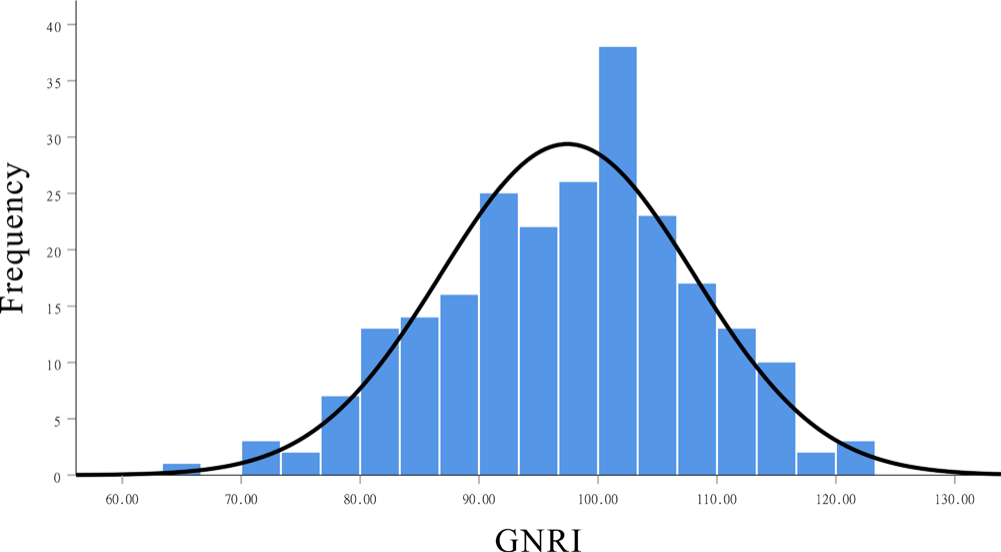
The GNRI exhibited a normal distribution. GNRI = geriatric nutritional risk index.

The GNRI exhibited a normal distribution (Fig. 2). The mean GNRI was 97.4 ± 10.6, and the number of patients in the risk group and no risk group was 111 (47.2%) and 124 (52.8%), respectively. As shown in Table 1, some clinical characteristics tended to be different among patients grouped by GNRI. Male patients were more likely to have nutritional risks than female (*P* = .038). However, patients had hypertension had lower risk faced with nutritional risks (32.4% vs 50%, *P* = .046). BMI index was consistent with the GNRI in terms of nutritional status of the patient (*P* < .001). The lower GNRI was in patients with ascites than patients without ascites (*P* = .048). The larger the tumor size, the lower the GNRI (54.2% vs 38.5%, *P* = .016). In addition, the number of people with anemia was lower in the no GNRI risk group (13.7% vs 44.1%, *P* < .001), and patients with CTP class B was higher for patients with GNRI < 98 (54.1% vs 15.3, *P* < .001).

### 3.3. The Postoperative Survival

**Figure 3.**
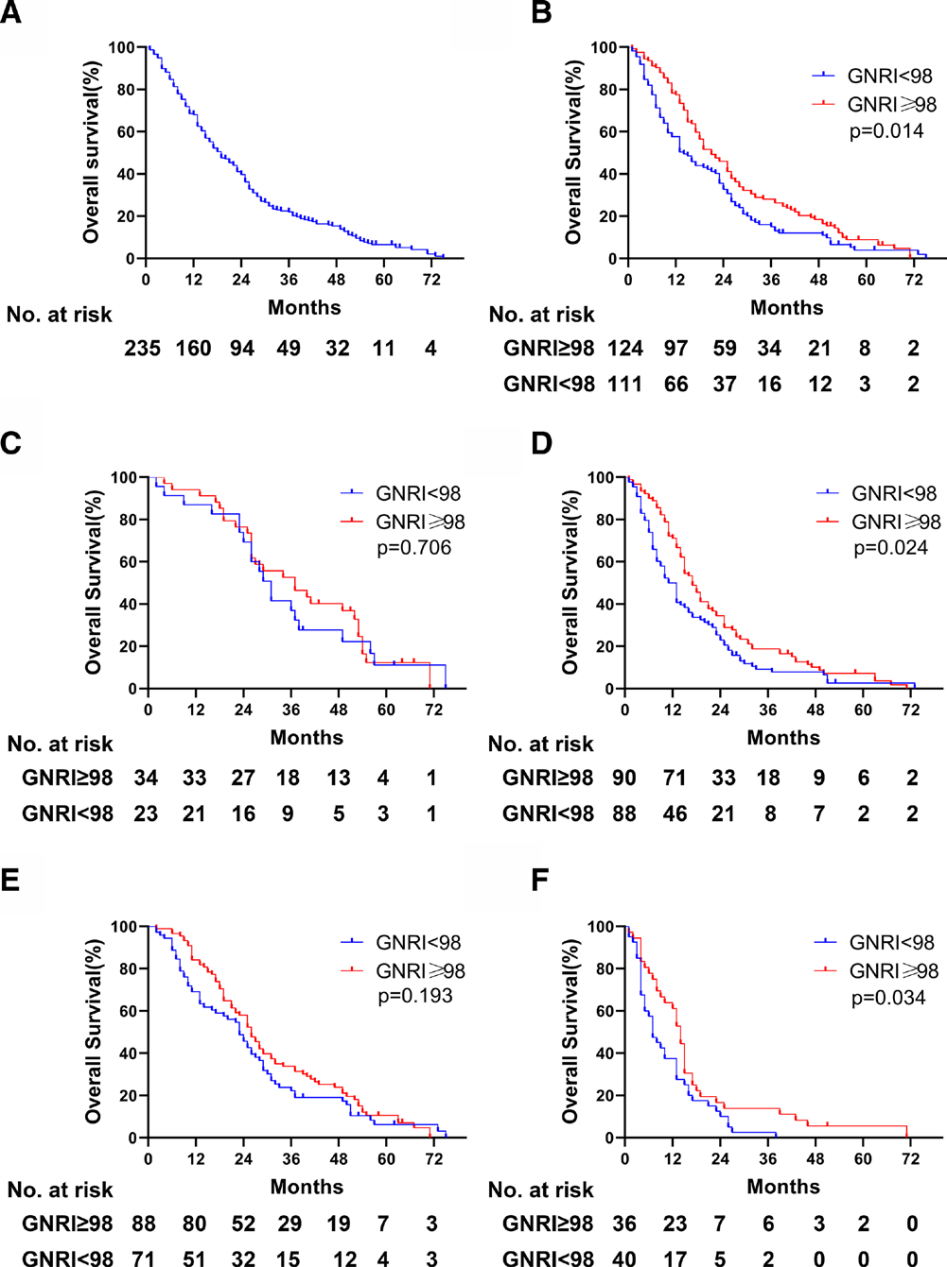
Kaplan-Meier estimates of overall survival (OS) for patients with the GNRI ≤ 98 vs GNRI > 98. (A-B) OS of HCC patients received TACE. (C-D) OS of HCC patient with stage A or stage B-C. (E-F) OS of HCC patient with AFP ≥ 400 or < 400. AFP = alpha-fetoprotein, HCC = hepatocellular carcinoma, TACE = transarterial chemoembolization.

At the time of analysis, 218 (92.8%) patients had died, and 3 patients were lost to follow-up. The median OS of the 235 patients were 19.0 months and the 1-, 3- and 5-year OS rates were 61.8%, 39.7%, and 6.5%, respectively (Fig. 3A). The 1-, 3- and 5-year OS rate of the patients with GNRI < 98 was 57.7%, 32.7% and 3.9%, respectively. The 1-, 3- and 5-year OS rate of the patients with GNRI ≥ 98 was 77.4%, 46.0% and 8.8%, respectively (Fig. 3B). Patients with GNRI ≥ 98 had longer OS than the patients with GNRI < 98 (*P* = .014).

We next evaluated the prognostic impact of the GNRI depending on the tumor stage and alpha-fetoprotein (AFP) level. Among the patients with stage A, the median OS was 31 months in the GNRI < 98 group and 37 months in the GNRI ≥ 98 group, respectively (*P* = .706; Fig. 3C). Among the patients with stage B-C, the median OS was 12 months in the GNRI < 98 group and 17 months in the GNRI ≥ 98 group, respectively (*P* = .024; Fig. 3D). For patients has AFP ≤ 400ng/mL, the patients had similar OS with GNRI ≥ 98 and GNRI < 98 (*P* = .193; Fig. 3E). For patients has AFP > 400ng/ml, the patients had longer OS with GNRI ≥ 98 than patients had a lower GNRI (*P* = .034; Fig. 3F).

### 3.4. Prognostic Factor Analysis

In the univariate analysis, the hazard ratio (HR) for GNRI ≥ 98 was 0.721 (95% CI 0.551–0.943, *P* = .017). The other factors correlated with the OS were tumor size (*P* < .001), vascular invasion (*P* = .019), AFP level (*P* < .001), BCLC stage (*P* < .001) and TACE times (*P* = .009). The multivariate analysis revealed that GNRI < 98 (*P* = .036), tumor size ≥ 5cm (*P* < .001), tumor number ≥ 2 (*P* = .037), AFP level ≥ 400 (*P* < .001) and TACE times < 3 (*P* = .004) were independent predictors of a poor OS (Table 2).

**Table 2:** Univariate and multivariate analyses of prognostic factors for HCC patients received TACE.

Variables		Univariate analysis			Multivariate analysis		
		HR	95%CI	*P* value	HR	95%CI	*P* value
Age (yrs)	<65 vs ≥ 65	0.867	0.653–1.151	.322			
Sex	Male vs female	1.188	0.851–1.657	.312			
Hypertension	Yes vs No	0.928	0.645–1.334	.686			
Diabetes mellitus	Yes vs No	0.901	0.593–1.370	.626			
Ascites	Yes vs No	1.404	0.783–2.517	.255			
BMI	<24 vs ≥ 24	0.781	0.597–1.023	.073	0.962	0.708–1.306	.804
Tumor size (cm)	<5 vs ≥ 5	2.388	1.809–3.151	**<.001**	2.105	1.558–2.844	**<.001**
Tumor number	<2 vs ≥ 2	1.219	0.911–1.633	.183	1.388	1.021–1.887	**.037**
Vascular invasion	Yes vs No	2.069	1.124–3.808	**.019**	1.532	0.816–2.883	.184
AFP level (ng/ml)	<400 vs ≥ 400	2.587	1.937–3.456	**<.001**	1.961	1.433–2.682	**<.001**
Anemia	Yes vs No	0.880	0.651–1.191	.408			
CTP class	A vs B	1.085	0.816–1.441	.575			
BCLC stage	A vs B–C	2.372	1.707–3.295	**<.001**	1.423	0.948–2.134	.089
TACE times	<3 vs ≥ 3	0.648	0.468–0.897	**.009**	0.612	0.440–0.851	**.004**
GNRI	<98 vs ≥ 98	0.721	0.551–0.943	**.017**	0.747	0.569–0.981	**.036**

AFP = alpha-fetoprotein, BCLC = barcelona clinical liver cancer, BMI = body mass index, CTP = child-turcotte-pugh, GNRI = geriatric nutritional risk index, HCC = hepatocellular carcinoma, TACE = transarterial chemoembolization.

### 3.5. Construction and validation of the nomogram

**Figure 4.**
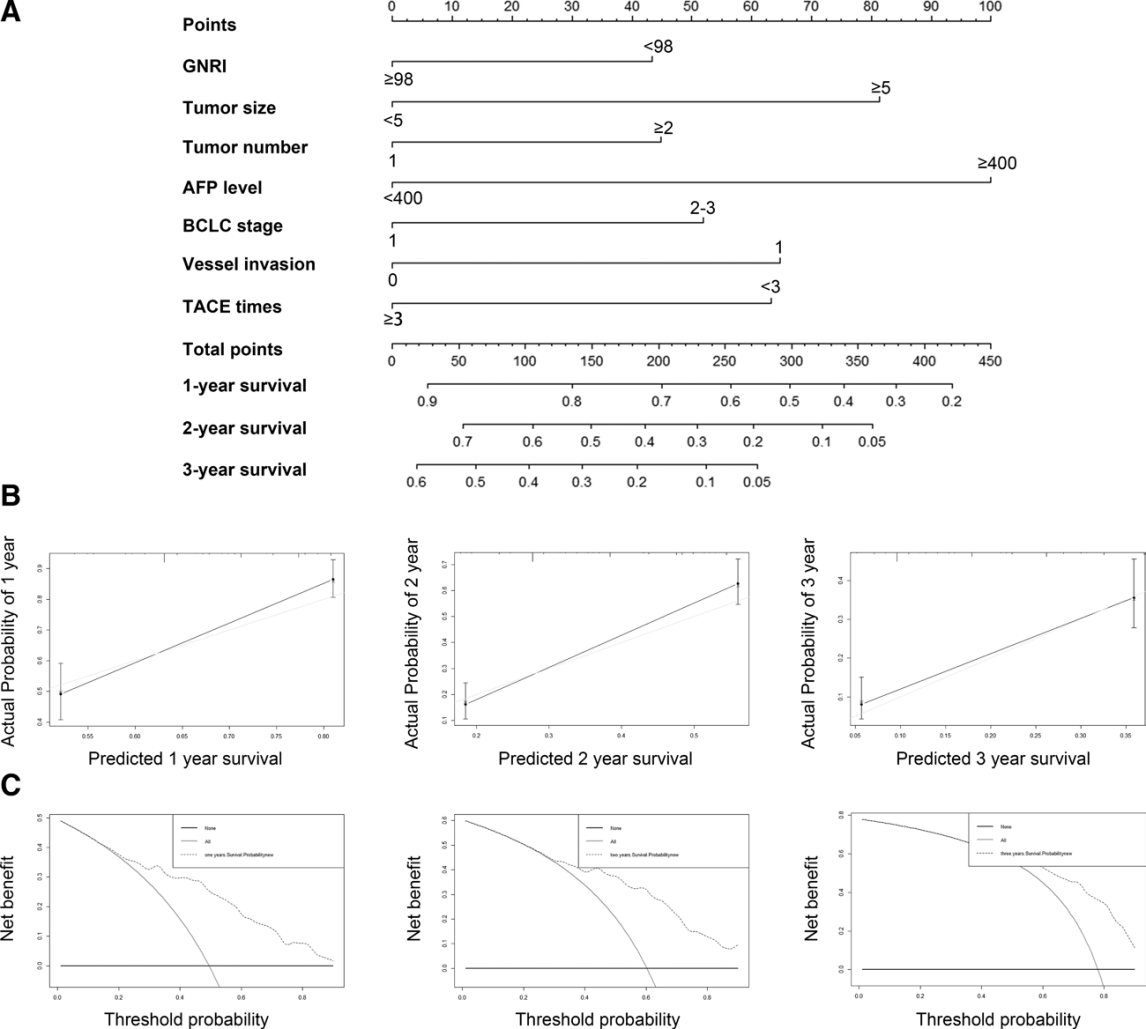
Nomogram, calibration plot and decision curve analysis of the nomogram for 3- and 5- year survival. (A) Nomogram to predict the survival time of HCC patients. (B) Calibration curves for predicting 1-, 2- and 3-year OS for HCC patients after TACE. (C) Time-dependent decision curve analysis for the clinical benefit of the nomogram. HCC = hepatocellular carcinoma, TACE = transarterial chemoembolization.

The C-index was used to predict the ability of the baseline nomogram, and which was 0.728 (95% CI: 0.695–0.753), demonstrating good fit for the model. The nomogram predicting the OS of HCC patients is displayed in Figure 4A, the 1-, 2- and 3-year survival probabilities were measured. The nomogram was well calibrated and got favorable overlap with the reference line, demonstrating the good performance of the model (Fig. 4B). Finally, we plotted the DCA in order to put these results in a clinical context. The combined model offered the best clinical utility from our prediction model (Fig. 4C).

## 4. Discussion

GNRI was named as a predictor of nutrition related risk index of mortality that could be used in all patients, including elderly patients, which was changed according to the Lorentz formula.^[18]^ Recent studies have demonstrated that the GNRI score was shown to be independently associated with the prognosis of cancer patients.^[7,19–21]^ In this study, we initially demonstrated that patients with HCC received TACE showed different OS rates according to their GNRI, and furthermore correlated clinicopathologic and GNRI with OS in HCC. More importantly, the present study was the first to propose a nomogram for predicting the OS of patients with HCC received TACE in China. The nomogram and risk classification system were developed and validated to provide more individualized and accurate prediction of OS for HCC patients treated with TACE.

Previous studies have suggested that prognostic factors for HCC cancer received TACE include the tumor number, microvascular invasion status, tumor size, treatment allocation, performance status, AFP level, et cetera.^[22–24]^ Peng et al^[22]^ suggested that the AFP and micro-vessel invasion of initial tumor were significant prognostic factors for OS and disease-free survival, respectively. Cheng et al reported that performance status (ECOG: 0) was an independent prognostic significant factor for 86 octogenarians with HCC initially treated with TACE.^[23]^ In our present study, we also found that tumor size, tumor number, AFP level and TACE times were independent predictors of OS, this further confirms the effectiveness of the above risk factors in predicting the prognosis of patients. Unfortunately, owing to controversial conclusions of the studies and unconventional examination, they are still not widely used in clinical settings. In conversely, our present study revealed that a lower value of GNRI impairs OS after TACE for patients with HCC, this provides a simple method for predicting the survival of patients with HCC after TACE.

Malnutrition is associated with worse outcome of cancer treatment, and body mass index (BMI) was the longest used to measure a patient’s nutritional status. Yu et al reported that not only low BMI but also high BMI patients were all had higher postoperative morbidity, including a higher incidence of surgical site infection after hepatectomy for HCC patients.^[25]^ However, Cha et al^[26]^ found that high BMI patients (BMI: 25–29.9 kg/m^2^) had better OS after TACE than normal BMI in males; HCC patients received TACE treatment resulted in significantly better OS in normal BMI patients than overweight in females. The different prognosis may be related to more than sex. Wu et al also reported that high BMI is associated with significantly more residual disease, new lesions, and progressive disease in patients with HCC treated by TACE.^[27]^ Thus, the influence of BMI on the survival after TACE has shown controversial results, new prognostic indicators need to be explored. The BMI consists of body weight and height, which are included in GNRI calculations,^[7]^ and serum albumin level is often reduced in HCC patients.^[28]^ Hypoalbuminemia has been proven to be a poor prognostic factor for a variety of malignancies,^[29]^ the GNRI may more objectively reflect the weight change in tumor patients due to tumor consumption, hence we consider that it is necessary to assess the patient’s nutritional condition using GNRI and assess the influence of GNRI on prognosis on HCC patients receiving TACE.

In this study, the multivariate analysis demonstrated that the risk groups (GNRI: < 98) was independent prognostic factor for HCC patients received TACE. Li et al^[10]^ reported that preoperative GNRI could predict severe postoperative complications, and the lower GNRI value was associated with worse OS after surgery treatment in elderly HCC patients. Sasaki et al^[9]^ found that low preoperative GNRI (≤98) was associated with increased postoperative complications and poor prognosis, preoperative GNRI can be used as a predictive indicator for potential high risk group of mortality in elderly colorectal cancer patients. However, a systematic review and meta-analysis founded that the GNRI was not an independent factor affecting cancer-specific survival, even though the results suggested that a lower GNRI was positively associated with worse OS of patients with gastrointestinal cancer.^[30]^ Therefore, the predictive value of GNRI in predicting the treatment outcomes of cancer patients needs to be evaluated further, in order to determine the appropriate population of cancer patients.

The mechanism of the value of GNRI on survival of cancer patients remains unclear and is has not been widely discussed. The authors consider that these mechanisms involve aspects of treatment for cancer in addition to immunology.^[7]^ Sonehara et al reported that higher GNRI was associated with better survival among patients with previously treated non-small cell lung cancer who were treated with immune checkpoint inhibitors, which might be related to that group having lower serum albumin levels.^[31]^ We think that the presence of nutritional decline may not be suitable for TACE treatment again for poor nutritional status may be associated with hypoproteinemia and massive ascites. In addition, a prospective study shows that a low GNRI value was associated with a poor prognosis and a low response rate to chemotherapy for extensive stage disease small cell lung cancer,^[21]^ it supports the relationship between systemic inflammation, nutritional status, and clinical outcomes.

The current study has several limitations. First, this was a retrospective single-center study, potential information and selection biases cannot be denied. Second, most of the patients in the study were not within the the definition of the elderly population by the GNRI. Third, GNRI was not compared with other commonly utilized tools to assess nutritional status, which should be assessed in future studies.

In conclusion, the present study suggests that low GNRI could predict bad prognosis for HCC patients received TACE. The GNRI can be used as a rapid low cost indicator. We believe that the GNRI should be used to assess patients with HCC in order to confirm nutritional status and estimate the prognosis as a convenient and efficient way.

## 5. Conclusion

Hepatocellular carcinoma (HCC) is the second cause of cancer-related deaths globally and over 50% of the new patients occur in China. Chemoembolization is the main treatment method. The nutritional status is of high significance for the patients’ performance status, the tolerance of tumor-targeting therapy and the prognosis of cancer of any type and is specially referenced in HCC. In the present study, we found the relationship between GNRI and prognosis in patients with HCC after TACE. The GNRI can be used as a predict indicator because of which is a rapid and low-cost method. We believe that the GNRI should be used to assess patients with HCC in order to confirm nutritional status and estimate the prognosis as a convenient and efficient way.

## Author contributions

**Data curation:** Aihua Xu.

**Formal analysis:** Aihua Xu.

**Methodology:** Peng Xu.

**Supervision:** Peiyuan Wang.

**Visualization:** Peiyuan Wang.

**Writing – original draft:** Youjiao Si.

**Writing – review & editing:** Kaikai Zhao.
